# Metastatic lymph node in gastric cancer; Is it a real distant metastasis?

**DOI:** 10.1186/1471-2407-10-25

**Published:** 2010-01-29

**Authors:** Do Hyoung Lim, Hyeong Su Kim, Young Suk Park, Jeeyun Lee, Se Hoon Park, Ho Yeong Lim, Sang Hoon Ji, Min Jae Park, Seong Yoon Yi, Ji Yeong An, Tae Sung Sohn, Jae Hyoung Noh, Jae Moon Bae, Sung Kim, Cheol Keun Park, Won Ki Kang

**Affiliations:** 1 Division of Hematology and Oncology, Department of Medicine, Samsung Medical Center, Sungkyunkwan University School of Medicine, Seoul, Korea; 2 Division of Hematology and Oncology, Department of Internal Medicine, Hallym University Medical Center, Hallym University College of Medicine, Seoul, Korea; 3 Department of Surgery, Samsung Medical Center, Sungkyunkwan University School of Medicine, Seoul, Korea; 4 Department of Pathology, Samsung Medical Center, Sungkyunkwan University School of Medicine, Seoul, South Korea; 5 Current address: Department of Surgery, Yonsei University College of Medicine, Seoul, Korea

## Abstract

**Background:**

Currently, the TNM staging system is a widely accepted method for assessing the prognosis of the disease and planning therapeutic strategies for cancer. Of the TNM system, the extent of lymph node involvement is the most important independent prognostic factor for gastric cancer. The aim of our study is to evaluate the survival and prognosis of gastric cancer patients with LN#12 or #13 involvement only and to assess the impact of anatomic regions of primary gastric tumor on survival in this particular subset of patients.

**Methods:**

Among data of 1,008 stage IV gastric cancer patients who received curative R0 gastrectomy, a total of 79 patients with LN#12 (n = 68) and/or #13 (n = 11) were identified. All patients performed gastrectomy with D2 or D3 lymph node dissection.

**Results:**

In 79 patients with LN#12/13 involvement, the estimated one-, three- and five-year survival rate was 77.2%, 41.8% and 26.6% respectively. When we compared the patients with LN#12/13 involvement to those without involvement, there was no significant difference in OS (21.0 months vs. 25.0 months, respectively; P = 0.140). However, OS was significantly longer in patients with LN#12/13 involvement only than in those with M1 lymph node involvement (14.3 months; P = 0.001). There was a significant difference in survival according to anatomic locations of the primary tumor (lower to mid-body vs. high body or whole stomach): 26.5 vs. 9.2 months (P = 0.009). In Cox proportional hazard analysis, only N stage (p = 0.002) had significance to predict poor survival.

**Conclusion:**

In this study we found that curatively resected gastric cancer patients with pathologic involvement of LN #12 and/or LN #13 had favorable survival outcome, especially those with primary tumor location of mid-body to antrum. Prospective analysis of survival in gastric cancer patients with L N#12 or #13 metastasis is warranted especially with regards to primary tumor location.

## Background

In Korea, gastric cancer is one of the most common causes of cancer-related death [1]. Currently, the tumor, node, metastasis (TNM) staging system is a widely accepted method for assessing the prognosis of the disease and planning therapeutic strategies [2]. Of the TNM system, the extent of lymph node involvement is the most important independent prognostic factor for gastric cancer [3]. These prerequisites were taken into account in the new TNM classification established in 2002 by the Union Internationale Contra le Cancer (UICC) and American Joint Committee on Cancer (AJCC). The 4th N-classification was based on the sites of lymph node metastasis (less than or greater than 3cm from the primary tumor) [4,5], whereas in 5th (1997) and 6th (2002) TNM editions, the N staging was based on the number of metastatic lymph nodes [6-9].

In the 6th edition of AJCC TNM classification [7], however, metastasis to intra-abdominal lymph nodes, such as hepatoduodenal, retropancreatic, mesenteric, and para-aortic, are still categorized as distant metastases. In support of this, Roder et al also categorized hepatoduodenal ligament lymph node involvement as distant metastasis [10]. In Japanese Gastric Cancer Association (JGCA) N-classification, every single lymph node was numbered as station (#1 to #112) and grouped by anatomical position [11]. According to the Japanese classification, hepatoduodenal lymph node is further numbered as station 12 (#12) and sub-classified as #12a (left hepatoduodenal lymph node) and #12b, p (posterior hepatoduodenal lymph node). Any lymph node stations greater than #12b are considered group 3 or distant metastases, and subsequently being categorized as stage IV gastric cancer.

Despite of such classification, several studies have demonstrated favorable survival in subsets of patients with lymph node metastases only. Chung et al. reported favorable outcomes of 5-year survival reaching 47.2% in a subgroup of gastric cancer patients with lymph node #12 to #14 metastases only [12], which is considerably higher than those reported for the historical control [13,14]. One of the plausible explanations for favorable survival in this particular group of patients may owe to different lymphatic drainage system depending on varying anatomic sites of the stomach. Upper third lymphatic vessels drain along left gastric, posterior gastric and splenic artery; whereas the lower third drains via common hepatic and superior mesenteric artery. Middle third stomach has a mixed drainage in both ways. All these vessels are eventually connected to the para-aortic lymphatic network [15-17]. Hence, the anatomic site of gastric cancer may be important when categorizing lymph node stations as distant metastases.

The aim of our study is to evaluate the survival and prognosis of gastric cancer patients with LN#12 or #13 involvement only and to assess the impact of anatomic regions of primary gastric tumor on survival in this subset of patients.

## Methods

We reviewed surgical records and pathologic data of 5,687 patients with gastric adenocarcinoma who underwent gastrectomies between January 1995 and December 2002 at Samsung Medical Center. All of the included patients were restaged according to the 6^th ^edition of AJCC and UICC [7,9]. In addition metastatic lymph node stations were classified according to the 2^nd ^English edition of Japanese classification of gastric carcinoma [11]. Among these, curative R0 resection was performed in 1,008 stage IV patients including 79 patients with pathologically confirmed hepatoduodenal lymph node involvement (LN#12, n = 68) and/or LN#13 (n = 11) only. The outcomes of the 1,008 stage IV patients who received curative gastric resection will be reported elsewhere: in brief, the median age was 57 years (range, 25-75 years), and the estimated median overall survival (OS) was 20.1 months. All patients received gastric resection and D2 or D3 lymphadenectomy. While our department policy usually recommends removal of LN#12 and #13, pathologic examination of LN#12a from the other nodes of the hepatoduodenal ligament was seldom performed. Macroscopic findings of tumor and microscopic tumor growth patterns were described by Bormann type and Lauren classification, respectively.

Written informed consent was given by all patients prior to surgery according to institutional guidelines, and the study was approved by the Samsung Medical Center (Seoul, Korea) institutional review board and any ethical approval was not required. After gastrectomy, two-thirds of the patients were treated with postoperative adjuvant chemotherapy (57%) or chemoradiotherapy (10%). While our department policy usually recommends adjuvant treatment if the tumor stages Ib to IV, decisions regarding postoperative treatment were individualized by the treating physician. Chemotherapy regimens were mostly cisplatin-based doublets, and chemoradiotherapy consisted of 45 Gy of radiation with leucovorin and 5-fluorouracil.

The starting point of OS was the day of gastric resection. Time to death, whatever the cause, was used to calculate OS. Investigation of the relationship between the lymph node status and OS was conducted using univariate and multivariate analyses. Survival curves and their confidence intervals (CI) were calculated according to the Kaplan-Meier method. The log rank test was used to assess the statistical differences between groups, and the Chi-square test was applied to assess differences in the distribution of patients among groups. To identify the factors that might be of independent significance in influencing the OS, Cox proportional regression model was fitted.

## Results

**Figure 1:**
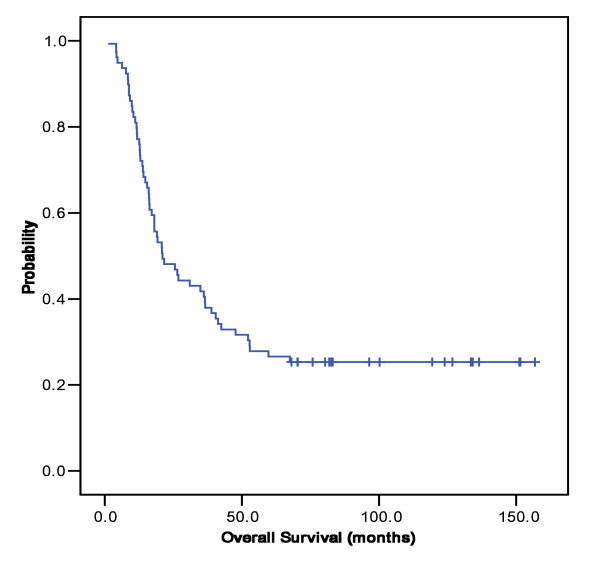
**Overall survival**. Kaplan-Meier overall survival curve. The median overall survival was 21.0 (95% C.I; 12.6-29.5) months.

**Figure 2:**
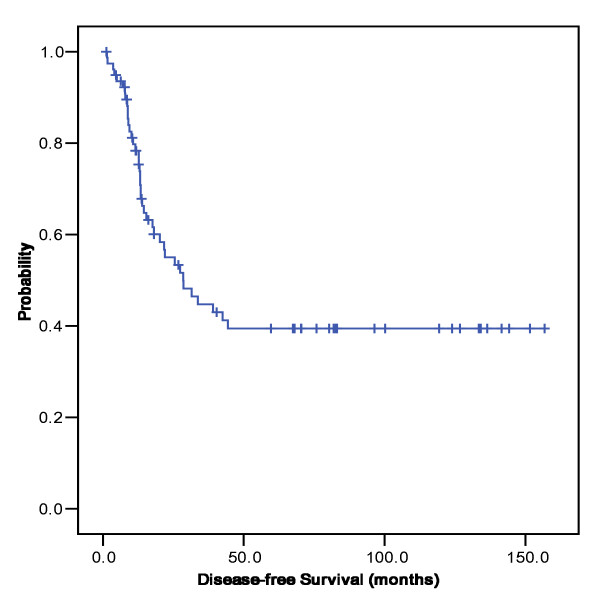
**Disease-free survival**. Kaplan-Meier disease-free survival curve. The median disease-free survival was 28.5 (95% C.I; 14.7 - 42.3).

When the 1,008 stage IV gastric cancer patients were divided according to LN#12/13 involvement only or others, there were no differences in the baseline characteristics including age, sex, histological grades and postoperative treatment. The demographic and histopathologic data of the 79 patients with lymph node #12/13 involvement are provided in Table 1. The most common site of primary tumor was antrum (63%), followed by mid-body (23%), upper body (11%), and the whole stomach (3%). Type of macroscopic finding was Bormann type III (66%) in two-thirds, and approximately half of the patients had diffuse type in Lauren classification. The distributions of T stage were as follows: T1 (0%), T2 (38%), T3 (52%), and T4 (10%). With a median follow-up duration of 30 months, the estimated median OS was 21.0 months (95% CI, 12.6-29.5 months). The patients had an estimated one-, three- and five-year survival rate of 77.2%, 41.8% and 26.6% respectively (Figure 1) and median disease-free survival was 28.5 months (Figure 2). When we compared the patients with LN#12/13 involvement to those without involvement (i.e., T4 and/or N3 disease), there was no significant difference in OS (21.0 months vs. 25.0 months, respectively; P = 0.140). However, OS was significantly longer in patients with LN#12/13 involvement only than in those with M1 lymph node involvement (14.3 months; P = 0.001).

**Table 1:** Patient characteristics

Clinical variables	Patient No (%)
Sex (Number)	
Male	53 (67.1)
Female	26 (32.9)

Age (years)	
Median	57
Range	25 - 75

ECOG performance status (Number)	
0	10 (12.7)
1	68 (86.1)
2	1 (1.3)

Bormann type (Number)	
I	0
II	17 (21.5)
III	52 (65.8)
IV	10 (12.7)

Lymph node dissection (Number)	
D2	76 (96.2)
D3	3 (3.8)

Lauren classification (Number)	
Intestinal	20 (25.3)
Diffuse	38 (48.1)
Mixed	4 (5.1)
Unknown	17 (21.5)

Histological grade	
Well to moderate	23 (29.1)
Poor to undifferentiated	56 (70.9)

T staging (Number)	
T1	0
T2	30 (38.0)
T3	41 (51.9)
T4	8 (10.1)

N staging (Number)	
N1	14 (17.7)
N2	25 (31.6)
N3	40 (50.6)

Metastatic lymph node station (Number)	
#12	69 (87.3)
#13	10 (12.7)

Post-operation treatment (Number)	
None	21 (26.6)
Chemotherapy	48 (60.8)
Chemo-radiation	8 (10.1)
Unknown	2 (2.5)

Primary site (Number)	
Upper body	9 (11.4)
Mid-body	18 (22.8)
Lower body	50 (63.3)
Whole stomach	2 (2.5)

**Figure 3:**
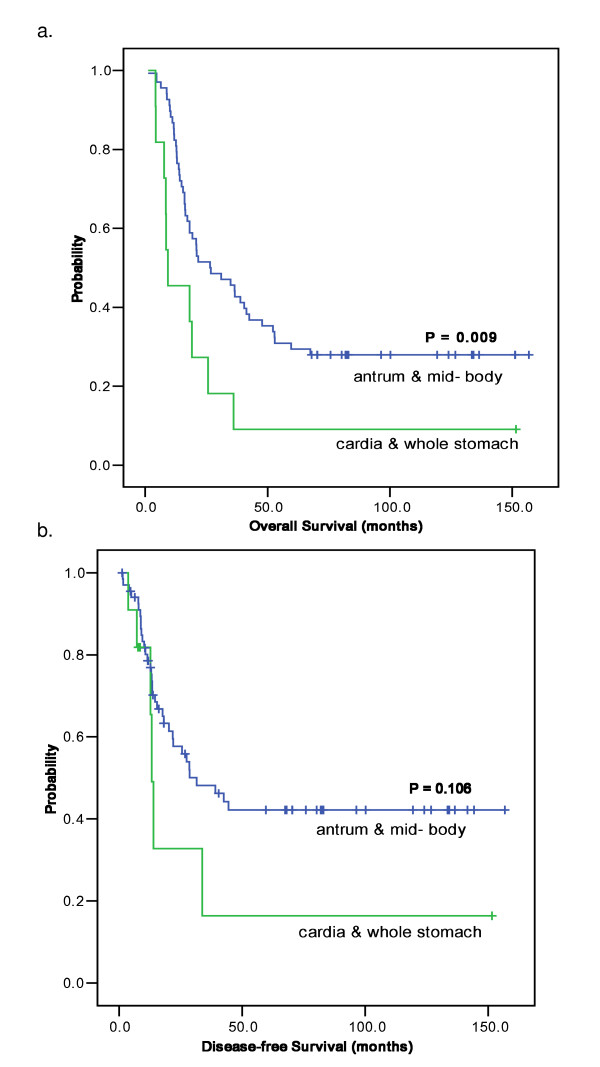
**Survival according to primary site**. **a. Overall survival according to primary site **The median overall survival of lower & mid-body was 26.5 (95% C.I; 10.6-42.3) months and that of high body & whole stomach was 9.2 (95% C.I; 0.0-19.7) months (p = 0.009). **b. Disease-free survival according to primary site **The median relapse free survival of lower & mid-body was 31.5 (95% C.I; 12.6-50.4) months and that of high body & whole stomach was 13.2 (95% C.I; 11.8-14.6) months (p = 0.106).

There was a significant difference in survival according to anatomic locations of the primary tumor (lower to mid-body vs. high body or whole stomach): 26.5 (95% C.I; 10.6 - 42.3) months vs. 9.2 (95% C.I; 0.0 - 19.7) months, respectively (P = 0.009) (Figure 3). However, there were no significant differences in clinical variables between the two tumor location groups except for types of surgery of the extent of lymph node dissection due to anatomic locations (Table 2). No significant difference in the distribution of N stage was observed (p = 0.066).

**Table 2:** Patients' characteristics according to primary sites

	Low & mid-body (n = 68)	High body & whole stomach (n = 11)	P value
Age (mean)	56.2	57.9	0.524

Sex			0.262
Male	44 (35.3%)	9 (81.8%)	
Female	24 (64.7%)	2 (18.2%)	

ECOG performance status			0.780
0	8 (11.8%)	2 (18.2%)	
1	59 (86.8%)	9 (81.8%)	
2	1 (1.5%)	0	

Bormann type			0.033
I	0	0	
II	16 (23.5%)	1 (9.1%)	
III	46 (67.6%)	6 (54.5%)	
IV	6 (8.8%)	4 (36.4%)	

Lauren classification			0.527
Intestinal	17 (25.0%)	3 (23.7%)	
Diffuse	31 (45.6%)	7 (63.6%)	
Mixed	4 (5.9%)	0	
Unknown	16 (23.5%)	1 (9.1%)	

Histological grade			0.390
Well to moderate	21 (30.9%)	2 (18.2%)	
Poor to undifferentiated	47 (69.1%)	9 (81.8%)	

Type of gastrectomy			< 0.001
Total gastrectomy	26 (38.2%)	11 (100%)	
Subtotal gastrectomy	42 (61.8%)	0	

Extent of surgery			< 0.001
Resection of spleen	9 (13.2%)	6 (54.5%)	
Resection of distal pancreas	2 (2.9%)	1 (9.1%)	
Resection of spleen, pancreas	0	2 (18.2%)	

T staging			0.543
T1	0	0	
T2	27 (39.7%)	3 (27.3%)	
T3	35 (51.5%)	6 (54.5%)	
T4	6 (8.8%)	2 (18.2%)	

N staging			0.066
N1	14 (20.6%)	0	
N2	23 (33.8%)	2 (18.2%)	
N3	31 (45.6%)	9 (81.8%)	

No. of dissected LNs (mean)	43.3	52.8	0.157

Metastatic lymph node station			0.701
#12	59 (86.8%)	10 (90.9%)	
#13	9 (13.2%)	1 (9.1%)	

Post-operation treatment			0.553
None	17 (25.0%)	4 (36.4%)	
Chemotherapy	41 (60.3%)	7 (63.6%)	
Chemo-radiation	8 (11.8%)	0	
Unknown	2 (2.9%)	0	

Lymph node dissection			0.007
D2	67 (98.5%)	9 (81.8%)	
D3	1 (1.5%)	2 (18.2%)	

In the univariate analysis applied to 79 LN#12/13 patients, OS was significantly longer in patients with low to mid body tumors (26.5 months) than in those with high body or whole stomach tumors (9.2 months, P = 0.012). Other factors associated with longer OS were Bormann type (I-II vs. III-IV, P = 0.031), N stage (p < 0.001), type of gastrectomy (p = 0.014), and extent of surgery (p = 0.006) (Table 3). In Cox proportional hazard analysis, only N stage (p = 0.002) retained its statistical significance to predict prognosis (Table 4). The anatomic location of primary gastric tumor did not significantly influence on survival of gastric cancer patients with LN#12 and/or LN#13 involvement at multivariate level (P = 0.906).

**Table 3:** Univariate analysis of prognostic factors for patients with metastatic lymph node positive gastric cancer

	1 YSR	3 YSR	Median OS (95% C.I^a^) months	H.R (95% C.I^a^)	P value
Age					0.461
< 65	82.1%	42.9%	25.6 (11.0 - 40.2)	1.0	
> 65	65.2%	39.1%	19.2 (11.8 - 26.6)	1.232 (0.707 - 2.145)	

Sex					0.204
Male	77.4%	47.2%	31.0 (15.2 - 46.9)	1.0	
female	76.9%	30.8%	16.1 (11.9 - 20.2)	1.416 (0.828 - 2.421)	

ECOG P.S					0.464
0	80.0%	40.0%	20.9 (0.0 - 49.6)	1.0	
1	76.5%	42.7%	21.0 (12.2 - 29.8)	0.776 (0.381 - 1.582)	
2	100%	0%	13.9 (13.9 - 13.9)	2.304 (0.285 - 18.624)	

Primary site					0.012
Low & mid-body	82.4%	45.6%	26.5 (10.6 - 42.3)	1.0	
High body & whole stomach	45.5%	18.2%	9.2 (0.0 - 19.7)	2.417 (1.216 - 4.806)	

Bormann type					0.031
I					
II	82.4%	58.8%	52.9	1.0	
III	78.9%	42.3%	21.7 (7.8 - 35.6)	1.845 (0.895 - 3.803)	
IV	60.0%	10.0%	13.0 (9.8 - 16.1)	3.521 (1.379 - 8.991)	

Lauren classification					0.207
Intestinal	75.0%	30.0%	16.3 (15.7 - 16.8)	1.0	
Diffuse	78.9%	57.9%	38.9 (31.0 - 46.8)	0.659 (0.344 - 1.233)	
Mixed	75.0%	25.0%	14.1 (0.0 - 29.0)	0.920 (0.266 - 3.181)	
Unknown	76.5%	23.5%	15.5 (12.3 - 18.7)	1.274 (0.622 - 2.610)	

Histological grade					0.954
Well to moderate	86.9%	39.1%	20.8 (16.4 - 25.2)	1.0	
Poor to undifferentiated	73.2%	42.9%	21.7 (5.9 - 37.5)	1.017 (0.578 - 1.787)	

Type of gastrectomy					0.014
Total gastrectomy	70.3%	29.3%	17.1 (13.2 - 21.1)	1.913 (1.143 - 3.203)	
Subtotal gastrectomy	83.3%	52.4%	36.6 (13.9 - 59.3)	1.0	

Extent of surgery					0.006
None	81.4%	49.2%	34.9 (16.4 - 53.4)	1.0	
Resection of spleen	60.0%	20.0%	13.0 (9.7 - 16.2)	2.937 (1.604 - 5.337)	
Resection of distal pancreas	66.7%	33.3%	16.1 (0.0 - 34.9)	1.353 (0.327 - 5.606)	
Resection of spleen, pancreas	100%	0%	18.1	1.953 (0.466 - 8.195)	

T staging					0.116
T1					
T2	80.0%	56.7%	38.9 (3.9 - 73.9)	1.0	
T3	75.6%	31.7%	19.1 (14.3 - 23.8)	1.797 (1.010 - 3.198)	
T4	75.0%	37.5%	16.1 (0.0 - 32.6)	1.861 (0.775 - 4.468)	

N staging					< 0.001
N1	92.9%	71.4%	59.6 (32.9 - 86.3)	1.0	
N2	88.0%	60.0%	52.2 (35.4 - 69.0)	1.121 (0.470 - 2.674)	
N3	65.0%	20.0%	14.8 (9.7 - 19.9)	3.870 (1.770 - 8.463)	

No. of dissected LNs					0.810
< 40	75.8%	42.4%	25.6 (4.7 - 46.6)	1.065 (0.637 - 1.781)	
> 40	78.3%	41.3%	20.8 (11.1 - 30.5)	1.0	

Metastatic lymph node station					0.230
#12	76.8%	39.1%	20.8 (12.2 - 29.4)	1.0	
#13	80.0%	60.0%	47.7 (0.4 - 95.0)	0.596 (0.256 - 1.388)	

Post-operative treatment					0.209
None	57.1%	38.1%	14.8 (5.4 - 24.1)	1.0	
Chemotherapy	83.3%	37.5%	21.0 (11.6 - 30.5)	0.869 (0.484 - 1.562)	
Chemo-radiation	100%	75.0%		0.266 (0.078 - 0.916)	
Unknown	50.0%	0%	11.6	1.020 (0.234 - 4.444)	

Lymph node dissection					0.357
D2	76.3%	43.4%	21.0 (8.3 - 33.8)	1.0	
D3	100%	0%	18.1 (15.2 - 21.0)	1.739 (0.536 - 5.643)	

^a^C.I; confidence interval

**Table 4:** Multivariate analysis of prognostic factors for patients with metastatic lymph node positive gastric cancer

Factor	Relative risk	95% C.I.^a^	P value
Type of gastrectomy	0.875	0.473 - 1.619	0.570
Extent of surgery	1.143	0.725 - 1.802	0.565
Bormann type	1.402	0.810 - 2.425	0.227
T staging	1.141	0.723- 1.801	0.570
N staging	2.033	1.305 - 3.167	0.002
Primary site	1.063	0.386 - 2.924	0.906

^a^C.I; confidence interval

## Discussion

In this study, we found that curatively resected gastric cancer patients with pathologic involvement of LN #12 and/or LN #13 had favorable survival outcome, especially those with primary tumor location of mid-body to antrum. To the best of our knowledge, there are no previous reports focusing on LN #12 and LN #13 involvements in gastric cancer. According to the UICC/AJCC 6th edition [7,9], hepatoduodenal lymph node (LN #12) and retropancreatic lymph node (LN#13) are categorized as distant (M1) metastatic lymph node. Although limited by inherent bias from retrospective analyses, our study showed that LN #12 and/or LN #13 pathologic metastases pursue discrete natural history apart from metastatic M1 disease [13,14]. Furthermore, there are several lines of evidence to support that gastric cancer with metastatic lymph nodes only have more favorable clinical outcome when compared to those with distant metastasis. Five-year survival rate of pN3 gastric cancer which was defined according to the 2002 AJCC staging system ranged between 10.5% and 13% in previous studies [18,19]. Based on our study, five-year overall survival rate of patients with LN#12 and/or LN#13 positive gastric cancer with primary tumor at antrum to body was 29.4%. These survival rate is comparable to that reported for curatively resected stage IIIA or IIIB gastric cancer as a historical cohort group [20]. Furthermore, we previously reported outcomes of postoperative treatment in gastric cancer to find the five-year survival rate was <15% in stage IV patients [21].

One of the plausible explanations for significant discrepancy in survival between antral and cardial stomach cancer with hepatoduodenal lymph node and retropancreatic lymph node may owe to different lymphatic drainage system. The antral stomach cancer cells may drain into hepatoduodenal lymph node at earlier stage when compared with those located at cardia for instance. The statistical significance of primary tumor site should be further validated in larger series of patients. Considering a marked survival discrepancy between the two groups, LN#12 or#13 metastasis should be further categorized according to primary tumor locations in future staging system.

Because lymph node metastases occur relatively early in gastric cancer, regional lymphadenectomy is the standard surgical procedure in addition to radical gastrectomy [1]. However, the extent of lymphadenectomy to achieve the optimal outcome is still controversial, and there is no worldwide consensus. Controversy exists regarding the extent of lymph node dissection, and whether it should be limited to the perigastric lymph nodes (D1) or include the regional lymph nodes outside the perigastric area (D2) [22]. The appropriate extent of lymph-node dissection for gastric cancer continues to be debated. Radical lymphadenectomy did not increase long-term survival after curative gastrectomy in either the landmark Medical Research Council trial [23] or in the Dutch [24] gastric trial. However, many proponents of radical lymphadenectomy report benefit of radical or extended radical lymphadenectomy. In a randomized trial comparing D1 to D2/D3 lymph node dissection, patients with D3 dissection showed an absolute overall survival benefit of 5.9% (95% CI; 7.3-19.1, log-rank p = 0.041) when compared with the control group [25]. Recently, another study demonstrated survival benefit of D2 or greater lymaphadenectomy over D1 surgery and concluded that given the nodal diffusion in their gastric cancer patients, extended lymphadenectomy is still a rationale to obtain radical resection [15].

Given a considerable difference in survival according to lymph node stations and primary tumor sites, extended lymphadenectomies may also be crucial in accurately staging the tumor. The LN#12 and higher stations are not generally removed in D1 dissection and in such case, there is a chance of misleading migration to a lower stage. Although criticized by postoperative complications in D2 or higher lymphadenectomies [23,24], recent studies showed no difference in the incidence of major complications or mortality between D1 and D2 dissections [26,27]. Notably, there was no significant difference in quality of life (QOL) after gastrectomy with D1 and D2/D3 lymphadenectomies [28]. Therefore, gastrectomy with extended lymphadenectomy may be beneficial not only in terms of survival but also accurate pathologic N staging.

This study is limited by small number of patients and intrinsic bias from retrospective analysis in nature. Our result should be interpreted with caution because it represents only a small group of patients with gastric cancer and the LN#12a and other lymph nodes of the hepatoduodenal ligament were not evaluated separately. Prospective evaluation of outcomes in subset of gastric cancer patients with LN#12 or #13 metastasis is definitely warranted especially with regards to primary tumor location.

## Conclusions

In this study, we found that curatively resected gastric cancer patients with pathologic involvement of LN #12 and/or LN #13 had favorable survival outcome, especially those with primary tumor location of mid-body to antrum.

## Competing interests

The authors declare that they have no competing interests.

## Authors' contributions

DHL, SHK and SHP drafted the manuscript. DHL, JL, SHP, SHJ, MJP and SYY collected the data and performed the statistical analysis. YSP, JL, SHP, HYL, JYA, TSS, JHN, JMB, SK and WKK followed the patients. CKP performed pathologic evaluations. YSP and WKK designed the study and helped with the manuscript. All authors read and approved the final manuscript.

## Pre-publication history

The pre-publication history for this paper can be accessed here:

http://www.biomedcentral.com/1471-2407/10/25/prepub
